# Skin Diseases and Syphilis

**Published:** 1896-04

**Authors:** 


### SKIN DISEASES AND SYPHILIS.

EDITED BY DR. M. B. HUTCHINS.

### SLEEP IN ITS RELATION TO DISEASES OF THE SKIN.

Dr. L. Duncan Bulkley, of New York, had a very valuable article on this subject, now reprinted, in *New York Medical Record* of November 20, 1895.

He sums up as follows:

"1. Sleep is an exceedingly important factor to consider in connection with many diseases of the skin, disorders in sleep occurring both as a contributing cause and as an effect of the same.

"2. The disorders of sleep occurring in patients with diseases of the skin may arise from many different conditions; the six principal causes may be classed as, *a*, digestive; *b*, toxic; *c*, circulatory; *d*, nervous (direct or reflex); *e*, psychic; and *f*, cutaneous.

"3. These causes of disturbances of sleep should be searched for and relieved, because of the injury resulting from imperfect sleep in producing or aggravating many diseases of the skin.

"4. In cases where the sleep disturbance is caused by the disease of the skin, the effort should be made to get relief to the insomnia by the proper internal and external treatment of the skin affection, before resorting to hypnotics; attention to details is often very necessary to secure this end.

"5. Preparations of opium may be resorted to when the disturbance of sleep is caused by pain connected with the skin disease, but these are useless or harmful when the wakefulness results from itching. Chloroform or ether are also not to be advised for this purpose.

"6. Some of the newer so-called anti-neuralgic and hypnotic remedies are often of great service in quieting the general irritation and inducing sleep, and gelsemium and cannabis indica are also valuable. It is often desirable to give repeated doses, at half-hour intervals, until the desired effect is produced."

### LEADWATER IN SKIN THERAPY.

(Dr. C. Boeck, *M. für Prak. Derm.*, xxi. No. 3.)

In inflammatory, severely itching, non-oozing skin diseases, the following very simple treatment is recommended:

℞ Talc. pulv.

Amyli.......................................... .aa ʒi gr. xl.

Glycerine.......................................... m. xl.

Aquæ plumbi.......................................℥iii *M.* xx.

*M.* Shake before using, add an equal quantity of cold water, so that it becomes very thin, and apply with an artist brush, or cloth, to the affected skin. Drying very rapidly, binding on is not necessary.

It is both a cooling wash and powder, and therefore to be recommended not only in acute, but also in many very itchy, chronic eczemas—especially of the anus and genitals. Contraindicated in "weeping" conditions.—*From Centralblatt für Chirurgie, No.* 8, '96.

---

### COLLES'S LAW IN A PROFESSIONAL NURSE.

Dr. Brocq also reports above case of Dr. H. Feulard. The woman's child had *hereditary* syphilis—absolutely no evidence of present or previous syphilis in the mother. Fournier later confirmed the diagnosis in the case of the child. Dr. Brocq remarks upon the mother having borne a syphilitic child, with no apparent symptom of the disease in herself, and her having nursed the child without contracting a mammary chancre. Dr. Feulard justly held that such a mother would be an unsafe wet-nurse, but safe for an already syphilitic infant, in which case she would be an ideal nurse. —*Journ. Cut. and Gen.-Ur. Dis., Jan.*, 1896.

---

### PSORIASIS TREATED BY MERCURIAL INJECTIONS.

Two cases of this disease are reported by Dr. J. Brault. Various attacks had been relieved by the usual remedies. In the last outbreak five centigrammes of yellow oxide of mercury were injected March 16th, 1895; four other injections by April 16th, and from May 9th to June 17th three hypodermics of ten centigrammes each.

On May 23d, all trace of eruption disappeared. The two cases were identical.—*Dr. Brocq's Paris Letter Journ. Cut. and Gen.-Ur. Dis., Jan.,* '96.

---

### EXTENSIVE CHANCROID—CLINIC CASE.

Case of prostitute. History of an operation for hemorrhoids about a year ago. Later the development of ulcerous lesions—"thought" by patient to be of cancerous nature. Examination revealed almost complete destruction of the perineum, and margins of the anus, right labium minor perforated, and an indefinite extension into the vaginal walls. Ulcerous surface large, granular edges sharp cut, soft. A sanious fluid oozing from vagina.—*M. B. H.*

